# Concordance between the schedule for the evaluation of individual quality of life-direct weighting (SEIQoL-DW) and the EuroQoL-5D (EQ-5D) measures of quality of life outcomes in adults with X-linked hypophosphatemia

**DOI:** 10.1186/s13023-022-02250-8

**Published:** 2022-02-23

**Authors:** Ravi Jandhyala

**Affiliations:** 1 Medialis Ltd, 13 Horse Fair, Banbury, OX16 0AH UK; 2 Centre for Pharmaceutical Medicine Research, Institute of Pharmaceutical Science, Faculty of Life Science and Medicine, King’s College University, London, UK

**Keywords:** XLH, Neutral theory, Quality of life, EQ-5D, SEIQoL

## Abstract

**Background:**

Accurate measurement of any constructs in clinical studies is of critical importance, especially if the adoption of an intervention relies on detecting a significant treatment effect where one exists. Under Neutral theory, the amount of relevant and irrelevant indicators selected to operationalise the construct contribute equally to the accuracy of the observation. The Neutral or accurate observation is achieved by observing all relevant indicators only. Generic QoL instruments such as EQ-5D are increasingly being accepted as imprecise, especially in rare diseases, based on the relevance of their indicators. QoL is a construct that embodies a patient's subjectivity, individuality, and local circumstances at measurement. SEIQoL-DW is an instrument designed to respect these characteristics of QoL through eliciting indicators or cues directly from the subject along with the proportion of the overall QoL they contribute. EQ-5D and SEIQoL can therefore be considered as being at opposing ends of accuracy in QoL measurement. XLH is a hereditary, progressive, rare disease characterised by phosphate wasting, affecting both children and adults and impacting their QoL. The purpose of this study was to observe if any change in QoL of adult XLH patients were detectable using EQ-5D, SEIQoL eliciting new cues at each visit, and SEIQoL administering baseline cues overall visits (thereby silencing its time-dependency) versus baseline over 12 months. In addition, any association between the three sets of observations was explored.

**Results:**

All quality of life scores were observed to decrease from baseline by 13.36%, 7.32% and 2.7% based on SEIQoL_visit_cues_, SEIQoL_baseline_cues_, and EQ-5D assessments, respectively. The decrease in the quality of life scores was only statistically significant (*p* = 0.037) for SEIQoL_visit_cues_. Beyond the baseline visit, the only highly positive and statistically significant pairwise association was between SEIQoL_visit_cues_ and SEIQoL_baseline_cues_ at M6 (*ρ* = 0.782, *P* value < 0.05) and M9 (*ρ* = 0.879, *P* value < 0.05).

**Conclusions:**

EQ-5D and SEIQoL_baseline_cues_ failed to detect the same statistically significant decrease in QoL observed by SEIQoL_visit_cues_. Both sets of SEIQoL observations were more closely associated with each other than with EQ-5D. Observing constructs such as QoL in rare diseases benefit from a Neutrality in indicator selection and respecting variation in dominance of various indicators over time.

## Introduction

X-linked hypophosphatemia (XLH) is a rare, hereditary, progressive, and lifelong disease associated with significant morbidity [1, 2] and a negative impact on the quality of life of the affected individual [3]. As an inherited disease [2], XLH affects both children and adults, resulting in life-long consequences across the lifespan [4]. Age and female sex are some of the characteristics associated with significantly impaired quality of life in adult XLH patients [3], with many of these issues originating in childhood [5].

While there have been significant advances in the available treatment options and management of adults with XLH [6, 7], ongoing concerns regarding their quality of life and the accurate measurement of this construct remain [8]. Adopting any new treatment option relies not only on favourable supporting evidence being made available promptly but also that this evidence answers the specific research questions of any relevant 'gatekeepers' of its progression to the patient. Recent research advocated a multiple stakeholder approach to real-world evidence generation [9] and modelled the adoption of a new medicine as an open system comprising three subsystems in series: the regulator, the payor, and finally, the prescriber [9]. Each subsystem requires specific evidence to satisfy its internal logic for that medicine to progress in its adoption. Evidence of quality of life improvements in rare disease patients receiving a particular treatment is valuable in payor discussions [10] and therefore an essential input for the Payor subsystem [11].

Given the fundamental importance of quality of life evidence in new medicine adoption, various authors have highlighted accuracy in developing these measurement instruments as a necessity [12, 13]. Evidence from Dowding et al. [14] suggests that generic quality of life measures may not effectively capture the impact of a specific disease, as they may be less sensitive to the condition, bringing into question how investigators select a reference against which they can assess the accuracy of their observations for a given construct. It is this question which forms the subject for Neutral theory [15]. The Neutral theory describes the construct of Neutrality (N_0_), or the accuracy, of observation of *any* given construct when measured against the reference of its true value, which an observer makes with complete accuracy or Neutrality. Making a Neutral or accurate observation relies on a Neutral list of indicators, for that construct, that the observer uses (a) exclusively and (b) without omission in their measurement of the construct. Should the observer deviate from either of these conditions, they reduce the Neutrality of their observation by reducing its sensitivity and specificity. Recent research applied Neutral theory and assessed the Neutrality of generic QoL instruments in diseases where disease-specific ones existed, using that latter as a surrogate for the Neutral list. The research concluded that *'Generic HRQoL tools appear poorly correlated with disease/condition-specific tools, which indicates that adoption of Neutral Theory in the development and assessment of HRQoL tools could improve their relevance, accuracy, and utility in economic evaluations of health interventions'* [12], pg. 1].

The source of the indicators used to observe the construct of quality of life of an individual must be specific to that individual, as the construct is inherently personal. This suggestion builds on evidence for the subjective nature of quality of life measures because the patient usually reports them [16]. However, generic measures may overlook these individual dynamics in quality of life, which explains the continued advocacy in the published literature for idiographic assessment in measuring the quality of life in patients [17]. By their design, idiographic assessments consider the individual nature of patients completing quality of life measures and often have a qualitative interview component in the data collection methods [17]. Ibrahim [18] argues that, as a research methodology, qualitative interviewing is sensitive to eliciting the required responses when assessing patients' subjective nature of quality of life. Essentially, QoL is subjective and individual, and approaches to its measurement need to respect these aspects if they are accurate.

The Schedule for the Evaluation of Individual Quality of Life -Direct Weighting (SEIQoL-DW), as an idiographic assessment, is an established method of exploring quality of life [19, 20] and has been used extensively among patients with rare and non-rare diseases [21, 22]. Compared to other generic tools such as the EuroQoL-5D (EQ-5D) [23], the SEIQoL-DW is an exact and fitting tool. Furthermore, SEIQoL-DW uses semi-structured interviews and judgment analysis to elicit direct weighing from patients on areas of their lives that are important and have been affected by their disease condition [20, 22].

The use of judgment analysis [24], culminating in the generation of nominated life areas (cues) relating to five key domains that the individual considers important, ensures that the quality of life measured by the SEIQoL is individual-focused. Furthermore, these cues serve as the basis for the direct weighting of quality of life, which is used as the benchmark during the subsequent quality of life assessments [19]. Researchers may administer SEIQoL to an individual serially over a period of time to monitor the effect of time on the SEIQoL index and its contributing domains, as well as their relative proportions.

Although both the EQ-5D and the SEIQoL-DW are quality of life (QoL) tools completed by the individual [22], cost-effectiveness modellers often use the EQ-5D as part of health technology assessment (HTA) submissions [25]. It is unclear whether there are similarities in the quality of life scores generated by these two instruments and if both tools can detect a change in the quality of life over time.

The author proposes instruments such as SEIQoL-DW and EQ-5D exist on a continuum of Neutrality, with individual interviewing at one extreme and generic questionnaires at the other. Given the empirical importance of accuracy in measuring any construct and specific risks associated with inaccurate quality of life measurements in medicine adoption, an assessment of the instruments sitting towards each extreme has been selected as the subject of this work. Furthermore, SEIQoL-DW offers the opportunity to assess an intermediate option by removing the time-dependent subjectivity through applying the baseline cues at each subsequent visit, thus resulting in the SEIQoL mimicking a fixed quality of life instrument.

### Study aim and objectives

This study aims to understand the comparative performance of the SEIQoL Index when applied using cues solicited at the Visit (SEIQoL_visit_cues_ Index) and when applied using cues solicited at baseline (SEIQoL_baseline_cues_ Index) and the EQ-5D instruments in measuring the quality of life in adult XLH patients over 12 months.

#### Objectives

The objectives are to:

1. Evaluate any change in QoL of adult XLH Patients over 12 months and at three-month intervals using EQ-5D, SEIQoL_baseline_cues_, SEIQoL_visit_cues_
2. Explore the concordance between the quality of life measured by EQ-5D, SEIQoL_visit_cues_, and SEIQoL_baseline_cues_ at baseline and each subsequent three-monthly visit.

## Methods

### Study participants and data collection procedure

The study initially recruited 11 patients from patient-led organisations to complete five assessments between August 2019 and February 2021. One participant withdrew after completing two evaluations, and the results presented report on the ten participants available for analysis at the end of the 12 months.

The study protocol required administration of EQ-5D, SEIQoL-DW at Baseline and three-monthly intervals after that up to one 1 year (M3,6,9,12). The SEIQoL-DW instrument applied cues elicited from the patients during the baseline assessment alongside the newly elicited cues at each subsequent visit. Each visit, therefore, generated three separate QoL observations.

### Statistical analyses

In order to compare the scores generated by the quality of life instruments, the EQ-5D scores were re-scaled from their original range of between − 0.59 and 1 to a range of 0–100 to match that of the SEIQoL-DW Index. The analysis plan used Spearman's rank correlation to assess the agreement between the EQ-5D and the SEIQoL-DW instruments for all baseline assessments. It also used the same test to determine the agreement in the change of quality of life as measured by the two instruments between consecutive visits, Baseline, and last visit (Baseline vs. M12). The analysis also involved using Wilcoxon signed-rank test to explore the statistical significance of any differences in quality of life scores between consecutive visits and between Baseline and the last visit (Baseline vs. M12). All statistical analyses were performed using R version 4.0.2.

## Results

Of the 11 participants recruited, 1 participant withdrew after two visits. Overall, 60% (6/10) of adult XLH patients included in the analysis were female, consistent with an X-linked dominant disorder. On entering the study, the youngest participant was 28 years old, while the oldest participant was 63 years old (Table 1). Although 90% (9/10) of study participants were employed, they reported that the XLH impacted their life and the 'Work' domain. All study participants were diagnosed with XLH in childhood. 9/10 were diagnosed at birth based on clinical presentation and family history. The one participant without a family history was diagnosed on clinical presentation alone. None of the participants had received genetic testing for XLH, consistent with the only recent availability of this technology.

**Table 1:** Demographic Characteristics and Clinical Profile of Study Participants (N = 10)

Characteristics	Participants n (%)
Gender (% Female)	6 (60)
*Age*	
Mean ± SD	46.1 ± 12.41
Range	28–63
Married/partner	5 (50)
Employed	9 (90)
Offspring with XLH	7 (70)
Family history of XLH	9 (90)
Diagnosed with XLH during childhood	10 (100)

The study participant with no known family history of XLH was therefore presumed to be a spontaneous case of XLH, which research suggests occurs in 20–30% of XLH cases [26].

The overall trend in the quality of life scores of adult XLH patients was observed to decrease from the Baseline towards the final visit, as measured by the two QoL instruments. Quality of life scores were observed to decrease by 13.36%, 7.32%, and 2.7% based on SEIQoL_visit_cues_, SEIQoL_baseline_cues_, and EQ-5D assessments respectively. This decrease in the quality of life scores was statistically significant (*p* = 0.037) for SEIQoL_visit_cues,_ only.

**Fig. 1:**
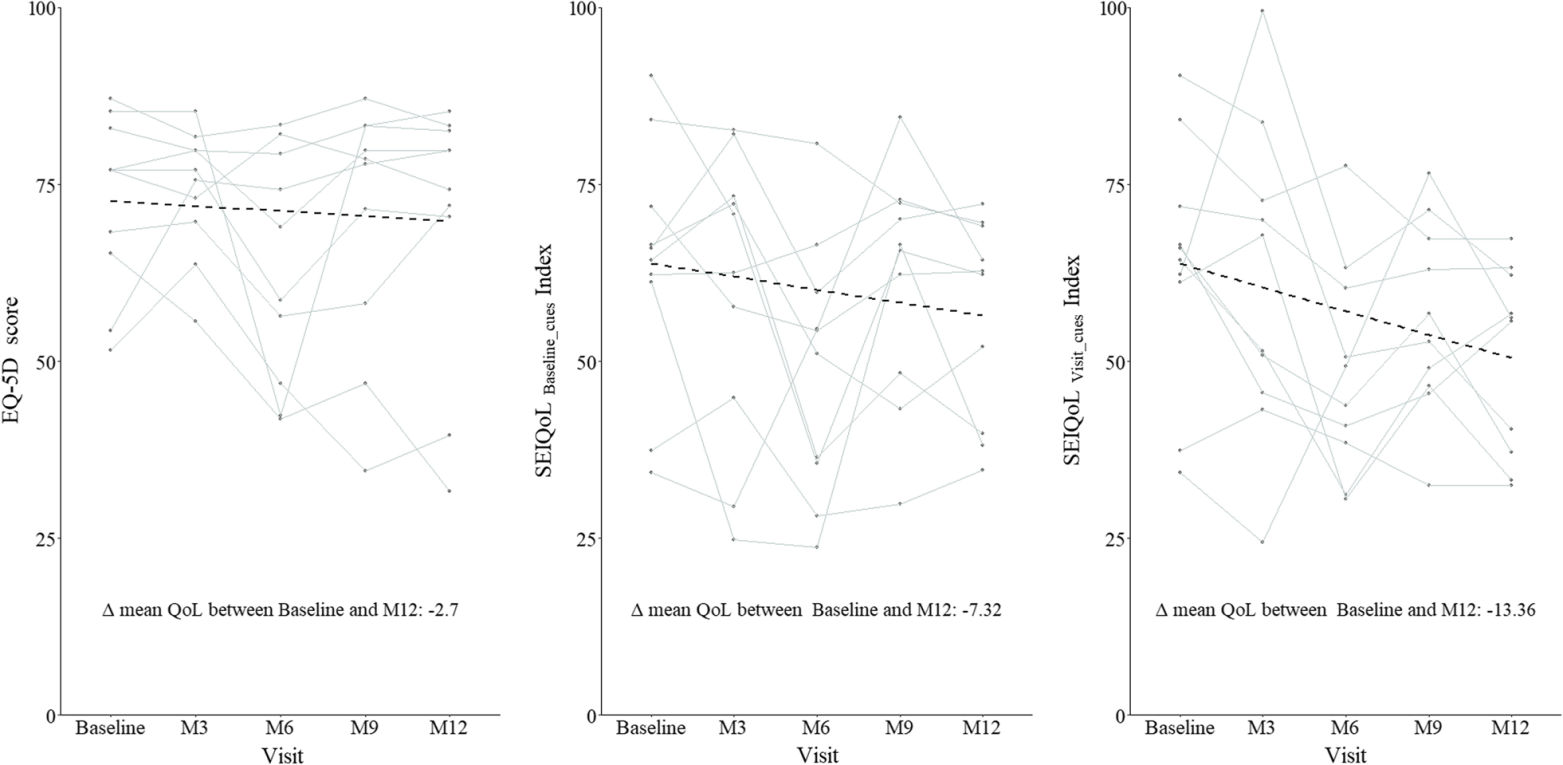
Dashed black line indicates the change in mean QoL between baseline and M12. Delta is the difference in mean QoL between baseline and M12. The values indicate the direction and magnitude of change along with the p-value indicating whether this change was significant

Table 2 provides the Wilcoxon signed-rank test result comparing the quality of life scores between the consecutive visits and between the Baseline and M12 visit assessment. Non-zero mean differences indicate improvement or deterioration of quality of life due to XLH between two points. The change in the quality of life among adult XLH patients in the study in consecutive time points is displayed in Fig. 1.

**Table 2:** Change in QoL of adult XLH Patients over 12 months and at 3-month intervals using EQ-5D, SEIQoL_baseline_cues_, SEIQoL_visit_cues_

Instrument	Visit comparison	Estimates of mean differences	*p*-value*
EQ-5D	M3 versus baseline	1.57	0.999
	M6 versus M3	− 10.73	0.037*
	M9 versus M6	6.68	0.105
	M12 versus M9	− 0.22	0.999
SEIQoL-DW (baseline cues)	M3 versus baseline	− 3.78	0.77
	M6 versus M3	− 10.95	0.131
	M9 versus M6	12.48	0.049*
	M12 versus M9	− 5.06	0.322
SEIQoL-DW (visit cues)	M3 versus baseline	− 2.89	0.275
	M6 versus M3	− 12.32	0.084
	M9 versus M6	7.52	0.084
	M12 versus M9	− 5.67	0.155
Overall	EQ-5D M12 versus EQ-5D baseline	− 2.7	0.375
	SEIQoL_baseline_cues_ M12 versus SEIQoL_baseline_cues_ baseline	− 7.32	0.232
	SEIQoL_visit_cues_ M12 versus SEIQoL_baseline_cues_ baseline	− 13.36	0.037*

^*^Statistically significant at 95% confidence interval

The pairwise relationship between QoL of adult XLH patients at each visit measured with SEIQoL_visit_cues_ and SEIQoL_baseline_cues_ and EQ-5D scores are shown in Table 3 below. There was a highly positive and statistically significant correlation in the observed quality of life scores at Baseline between SEIQoL-DW and EQ-5D assessments (ρ = 0.78, *P* value = 0.008). Beyond the baseline visit, the only highly positive and statistically significant pairwise association was between SEIQoL_visit_cues_ and SEIQoL_baseline_cues_ at M6 (ρ = 0.782, *P* value < 0.05) and M9 (ρ = 0.879, *P* value < 0.05).

**Table 3:** Concordance between the quality of life measured by EQ-5D, SEIQoL_visit_cues_, and SEIQoL_baseline_cues_ at baseline and at each subsequent three-monthly visit

Visit	Tools compared	Correlation coefficient	*p*-value*
Baseline	EQ5D versus SEIQoL	0.779	< 0.05*
M3	EQ5D versus SEIQoL baseline cues	− 0.012	0.973
M6	EQ5D versus SEIQoL baseline cues	0.042	0.919
M9	EQ5D versus SEIQoL baseline cues	− 0.158	0.663
M12	EQ5D versus SEIQoL baseline cues	0.006	0.987
M3	EQ5D versus SEIQoL visit cues	0.389	0.266
M6	EQ5D versus SEIQoL visit cues	− 0.018	0.973
M9	EQ5D versus SEIQoL visit cues	0.109	0.763
M12	EQ5D versus SEIQoL visit cues	0.517	0.126
M3	SEIQoL baseline cues versus SEIQoL visit cues	0.248	0.492
M6	SEIQoL baseline cues versus SEIQoL visit cues	0.782	< 0.05*
M9	SEIQoL baseline cues versus SEIQoL visit cues	0.879	< 0.05*
M12	SEIQoL baseline cues versus SEIQoL visit cues	0.527	0.123

^*^P-value indicating if the correlation coefficient is significantly different from the 0 value

Despite the observed correlation between the two instruments at Baseline, there are significant differences when comparing the scores generated using both instruments across the visits.

**Fig. 2:**
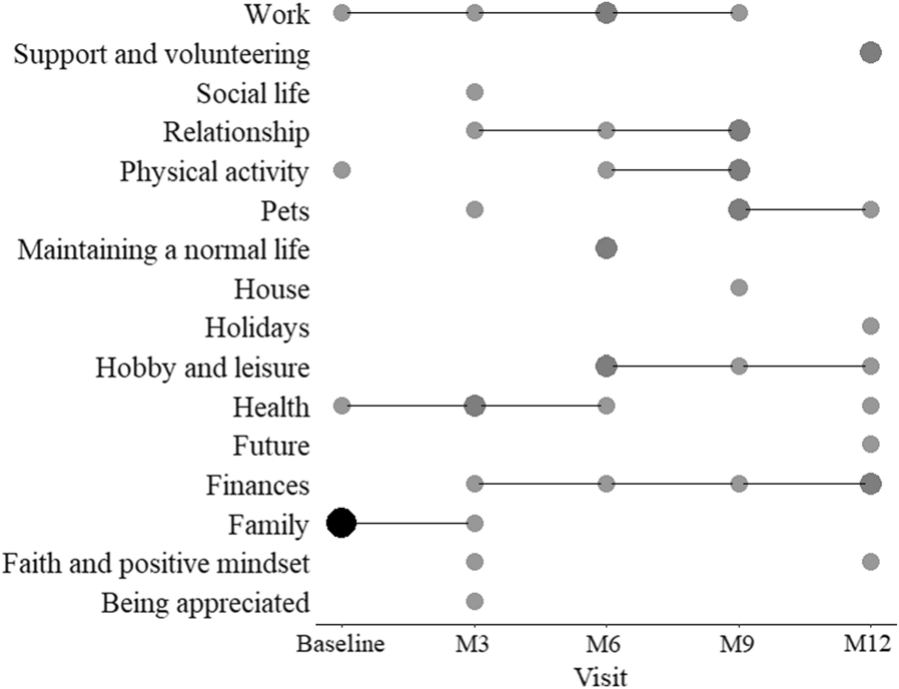
Cues elicited from XLH patients using SEIQoL-DW at each study visit. The size of the circles indicate the frequency of a cue at a given visit. Lines indicate if a cue appears in consecutive visits

The type and frequency of nominated life areas (cues) elicited using the SEIQoL-DW instrument across the visits in measuring the quality of life of adult XLH patients are shown in Table 4. Cues such as Family, Health, Work, Finances, Relationship, and Physical activity have a higher frequency and were suggested across multiple visits. At baseline, Family was the highest nominated life area. However, Family was not consistently identified as an important life area during subsequent assessments, particularly during assessments M3–M12. A plausible rationale for this could be linked to the underlying role of family members in supporting patients with rare diseases. Such that, while Family as a cue was not consistently nominated, many of the cues suggested by the patients were activities that would require the support of someone considered a family member (Fig. 2).

**Table 4:** Distribution of SEIQoL-DW cues from adult XLH patients over the five study visits

S/N	Cues	Baseline	M3	M6	M9	M12	Total
1	Being appreciated	0	1	0	0	0	1
2	Faith and positive mindset	0	1	0	0	1	2
3	Family	7	1	0	0	0	8
4	Finances	0	1	1	1	2	5
5	Future	0	0	0	0	1	1
6	Health	1	2	1	0	1	5
7	Hobby and leisure	0	0	2	1	1	4
8	Holidays	0	0	0	0	1	1
9	House	0	0	0	1	0	1
10	Maintaining a normal life	0	0	2	0	0	2
11	Pets	0	1	0	2	1	4
12	Physical activity	1	0	1	2	0	4
13	Relationship	0	1	1	2	0	4
14	Social life	0	1	0	0	0	1
15	Support and volunteering	0	0	0	0	2	2
16	Work	1	1	2	1	0	5
Discrete cues elicited at visit (n)		4	9	7	7	8	
Cues unique to the visit (n)		0	2	1	1	3

Although Health as a cue was not consistently nominated across all assessment visits, it was elicited at least once at 4 of the five visits. In addition, the results show a similar level of frequency with respect to the nomination of Work, Relationship, Finance and Physical activity, by study participants across the Visits. Overall, Family, Finances, Health and Work were the four most frequently elicited cues.

## Discussion

This research highlights a number of important considerations for how individuals with a disease are observed. The central principle espoused by Neutral theory is that efforts must be made to research and observe only those indicators relevant to a construct of interest. The consequences of deviating from this principle have been described both theoretically [15] and through reviewing published experience as a measurement of quality of life [12]. Accurate construct measurement in social sciences can be argued as being just as important as in natural sciences. However, given the key decisions made on the basis of findings from clinical studies particularly in the context of the adoption of new medicines [11] the consequences of inaccurate findings in this setting can be particularly harmful to the very patients the investigators are seeking to support.

Neutrality in indicator selection drives sensitivity and specificity of observation for the construct. In clinical studies, endpoints are defined by the instruments used in observing the subjects. This research compares, prospectively two extremes of Neutrality in quality of life measurement, EuroQoL-5D (EQ-5D) [23] and the Schedule for the Evaluation of Individual Quality of Life (SEIQoL) [19, 20]. The findings are consistent with the growing body of evidence on the inaccuracy of EQ5D, especially in rare diseases [12]. However, more concerning is the consequence in that a statistically significant change in the quality of life went undetected in this rare disease population and potentially unrecognised if it were not challenged with the concurrent use of SEIQoL-DW.

The inclusion of SEIQoL_baseline_cues_ to, remove the time-dependent variation in QoL indicator inclusion and mimic the behaviour of an intermediate instrument yielded the expected results in that the mean change in QoL observed with it lay between EQ5D and the pure SEIQoL-DW. A stronger association was seen between the two SEIQoL-DW instruments. This implies that even a moderate attempt at achieving Neutrality by using cues, accurate to at least one point in time, for an individual yields results closer to SEIQoL than EQ5D when administered concurrently. SEIQoL-DW, itself is inherently limited in the degree to which it can achieve Neutrality in observing QoL due to the limitation of eliciting only 5 cues and assuming their complete contribution to an individual's overall quality of life in subsequent direct weighting. These results show a total of 16 separate cues were solicited from the 10 participants over the course of the 12 months of the study. Though these cues lack a degree of granularity, their number exceeds those routinely observed through SEIQoL-DW.

It can therefore be suggested, via Neutral theory, that assuming all 16 domains or indicators are relevant to the measurement of adult XLH QoL and therefore included in the observation at each time point, an observation closer to Neutrality could be achieved and that this is realised through increasing the positive predictive value and therefore the sensitivity of the observation so reducing the false positive rate from when only using 5 of the 16.

The case for Neutrality in selecting indicators for any construct measurement has been made with specific reference to the quality of life in rare diseases here. However, this study also highlights the dynamic influence time has on the nature of the top five indicators contributing to the subjects' quality of life and therefore further impresses the need to encompass the totality of the possible indicators in any disease-specific fixed instrument. SEIQoL, respecting this time-dependent variation but only including a fraction of the possible cues, still detected an overall deterioration in the QoL of adult XLH patients over the 12-month duration of this study.

The highly positive and statistically significant correlation between EQ-5D and SEIQoL at baseline is difficult to explain. There is no structural methodological explanation for this finding which only leaves a chance correlation of the indicators observed across the two instruments at this visit. The subsequent loss of any agreement over the remaining visits can be attributed to the inflexibility of the EQ-5D to be able to learn from this previous event in the way SEIQoL_baseline_cues_ was modified to do and poor Neutrality in observing disease-specific QoL.

The limited responsiveness of the EQ-5D tool has been observed in other disease-specific conditions and has been shown to not adequately capture patients' experiences [27, 28]. According to Brettschneider et al. [27], the responsiveness of the EQ-5D tool was only observed in patients reporting better health, thereby suggesting that using the EQ-5D instrument to assess the quality of life of adult patients with rare diseases, a population likely to have worse health status, may be inappropriate.

Finally, considering the EQ-5D and SEIQoL on a continuum of Neutrality, and therefore the accuracy, in measuring QoL in adult XLH patients, EQ-5D remains on the least Neutral end of the spectrum and SEIQoL_visit_cues_ on the other with SEIQoL_baseline_cues_ occupying an intermediate position, if closer to its parent. The hypothesis, generated by this research and Neutral theory, that a disease-specific instrument including a Neutral list of indicators could be even more accurate than SEIQoLvisit_cues and would be a reasonable recommendation for further research in this group of patients.

Understanding the impact of rare diseases on the quality of life of adult patients continues to be an integral part of treatment and patient care. Focusing on adult XLH patients, underestimating the impact of the disease on quality of life may result in inadequate treatment availability, thus leading to worse health outcomes for these patients. While existing evidence has shown the importance of prioritising and giving credence to the quality of life of adult XLH patients [8, 29], the results here demonstrate accurate measurement of quality of life outcomes continues to be an issue if generic tools are used instead of disease-specific ones which exhibit greater Neutrality in their indicator selection.

The results here show that adult XLH patients do deteriorate in their QoL over a period of 12 months and that the statistical significance of this change was not detected by EQ-5D. Consequently, detection of statistically significant treatment effects of new interventions may similarly be undetected if observed by the EQ-5D in this population.

## Limitations

The generalisability of these results is subject to certain limitations. For instance, given that large sample sizes of rare disease patients are difficult to obtain, the sample size was small, and study participants were recruited from a patient-led organization. Hence, the views and experiences of adult XLH patients not belonging to the patient-led organization might not have been captured in this study. Another issue that was not addressed in this study was the impact that the COVID-19 pandemic might potentially have on the nominated life areas of study participants. Notwithstanding the relatively limited sample, this work offers valuable insights into the responsiveness of both the SEIQoL and EQ-5D tools in measuring the quality of life of adult patients living with a rare disease.

## Conclusions

This study had two key objectives. Firstly, it aimed to evaluate any change in the quality of life of adult XLH patients as measured by SEIQoL and EQ-5D tools over a 12 month period. It also aimed to explore the concordance in the change in quality of life scores between the two instruments between two consecutive visits and overall. The results of this study showed that EQ-5D is not an appropriate tool to monitor change in adult XLH patients over time. SEIQoL's Neutrality in indicator selection is limited to the 5 most important domains which change over time. The time-dependent variability can be artificially silenced within SEIQoL resulting in an overall finding closer to its parent instrument than EQ-5D but without reaching statistical significance.

## Data Availability

The datasets used and analysed during the current study are available from the corresponding author on reasonable request.
